# Association of handgrip strength weakness and asymmetry with low physical performance among Chinese older people

**DOI:** 10.1007/s40520-024-02886-5

**Published:** 2024-11-25

**Authors:** Yu Luo, Xiaoyi Ma, Ling Zhang, Wen He

**Affiliations:** Department of Geriatrics, The First Affiliated Hospital, Sun Yat-sen University, 58 Zhongshan 2nd Road, Yuexiu District, Guangzhou, Guangdong 510000 PR China

**Keywords:** CHARLS, Handgrip strength, Physical performance, Short physical performance battery

## Abstract

**Background:**

Handgrip strength (HGS) weakness and asymmetry are both abnormal conditions of upper-limb muscle strength. The association between HGS weakness and physical performance is controversial, and the link between HGS asymmetry and physical performance remains unclear.

**Aims:**

This study aimed to investigate the associations of HGS weakness and asymmetry separately and concurrently with low physical performance among Chinese older people.

**Methods:**

The study used two waves of data from China Health and Retirement Longitudinal Study (CHARLS) in 2013 and 2015. HGS weakness and asymmetry were defined according to the maximal HGS and the HGS ratio, respectively. Participants were classified into 4 groups according to HGS status: normal, asymmetry only, weakness only, and concurrent weakness and asymmetry. The logistic regression model was used to investigate the cross-sectional association between low physical performance and each of maximal HGS, HGS ratio, and HGS status, as well as the prospective association between baseline HGS status and new-onset physical performance decline after two years.

**Results:**

Participants with HGS asymmetry only, weakness only, and two abnormalities showed a higher prevalence of low physical performance when asymmetry defined as an HGS ratio exceeding 1.20 and 1.30 (all, *p* < 0.001), with the greatest odds in those with two abnormalities (20% threshold: OR 3.83; 30% threshold: OR 5.41). The longitudinal analysis found that HGS weakness can predict the new-onset low physical performance over a two-year period, with concurrent HGS asymmetry further increased the future risk of physical performance decline.

**Conclusions:**

Both HGS weakness and asymmetry were associated with a higher prevalence of low physical performance, in an additive way. This study will help screen older people with low physical performance more efficiently, and identify those at higher risk of developing new-onset physical performance decline within two years.

**Supplementary Information:**

The online version contains supplementary material available at 10.1007/s40520-024-02886-5.

## Introduction

Physical performance refers to the body’s capacity to manifest strength, endurance, speed, balance, and coordination in daily activities and exercise. The assessment of physical performance is a critical component in the evaluation of older people in both clinical and research settings, as it correlates with current health status and predicts future health outcomes [1–5]. A diverse array of tests, from single-item measures such as gait speed, various balance tests, Timed Up and Go Test (TUG), Chair Stand Test (CST), etc., to comprehensive assessment tools like the Short Physical Performance Battery (SPPB) [6, 7], are used to assess the physical performance of the older people. However, these tests are relatively complex and laborious, requiring trained staff, ample space, and sufficient time for implementation. Furthermore, explaining their procedures to the older people, particularly those with limited comprehension or cognitive impairments can pose challenges. Therefore, these assessment methods might not be practicable in all clinical settings and the necessity for simpler, more manageable measures becomes apparent.

Muscle strength is fundamental to physical performance, and handgrip strength (HGS) is the most widely used indicator of upper-limb muscle strength. Different from the measurement tools of physical performance we had mentioned above, HGS measurement is simpler, faster, and more cost-effective [8]. Weakness in HGS, defined as an HGS value below the established normal threshold, is one of the diagnostic criteria for sarcopenia and frailty [9–11]. However, the data on the association between HGS weakness and low physical performance are contradictory. A recent narrative review has highlighted the inconsistency within the extant literature regarding this correlation, calling for additional investigation [12]. One possible reason for this is that, while the muscle strength is crucial, it’s not the only determinant of physical performance in the older people. Muscular endurance, motor coordination, balance function, and other factors related to the nervous, muscular, and skeletal systems also play an important role in physical performance [13]. Furthermore, there is a lack of large-scale and representative data on Asian older adults in this research area.

HGS asymmetry, characterized by a noticeable difference in muscle strength between two hands, is recognized as another abnormal condition of HGS besides weakness [14, 15]. Increasing research focuses on the link between HGS asymmetry and adverse health outcomes in the older people. Notably, HGS asymmetry was associated with an increased risk of future falls [16], functional disability [17], activity limitations [15], and various diseases, including neurodegenerative disorders [18], cognitive decline [19], and stroke [20]. Considering the good correlation between upper- and lower-limb muscle strength [21], asymmetry in HGS could partially indicate a similar disparity in lower-limb strength. As a sign of impaired muscle function, bilateral strength asymmetry could negatively influence older people’s balance and mobility, potentially degrading their physical performance. However, only few studies have explored the relationship between HGS asymmetry and physical performance [22], and such data among Chinese older people is lacking.

To summarize, both weakness and asymmetry were abnormal statuses of HGS, with association between HGS weakness and physical performance being controversial, and the link between HGS asymmetry and physical performance remaining unclear. Both require further investigation. When HGS weakness and asymmetry occur together, they may indicate a higher risk of low physical performance than either condition alone, yet data on this topic is limited. To bridge these gaps, our study examined the cross-sectional and prospective associations of both HGS weakness and asymmetry, individually and collectively, with physical performance in Chinese older people. We utilized data from two waves of the China Health and Retirement Longitudinal Study (CHARLS), conducting an initial cross-sectional analysis in 2013 (wave2) and a subsequent longitudinal analysis that extended to 2015 (wave3). This study may help to simplify the process of physical performance assessment, facilitating early detection and prevention of physical performance decline in the older population.

## Methods

### Study population

CHARLS is a national, longitudinal investigation focused on individuals aged 45 years and older in China. From 2011 to the present, CHARLS has released five waves of data, in which information on demographics, health, economics, and social circumstances was collected. The CHARLS conducted a multi-stage probability sampling method to recruit participants, and the detailed descriptions of CHARLS have been published in previous literature [23]. Access to CHARLS datasets is facilitated through its official website at http://charls.pku.edu.cn/en. The CHARLS survey project was approved by the Biomedical Ethics Review Committee of Peking University (IRB00001052–11015). All participants signed informed consent before data collection.

**Fig. 1:**
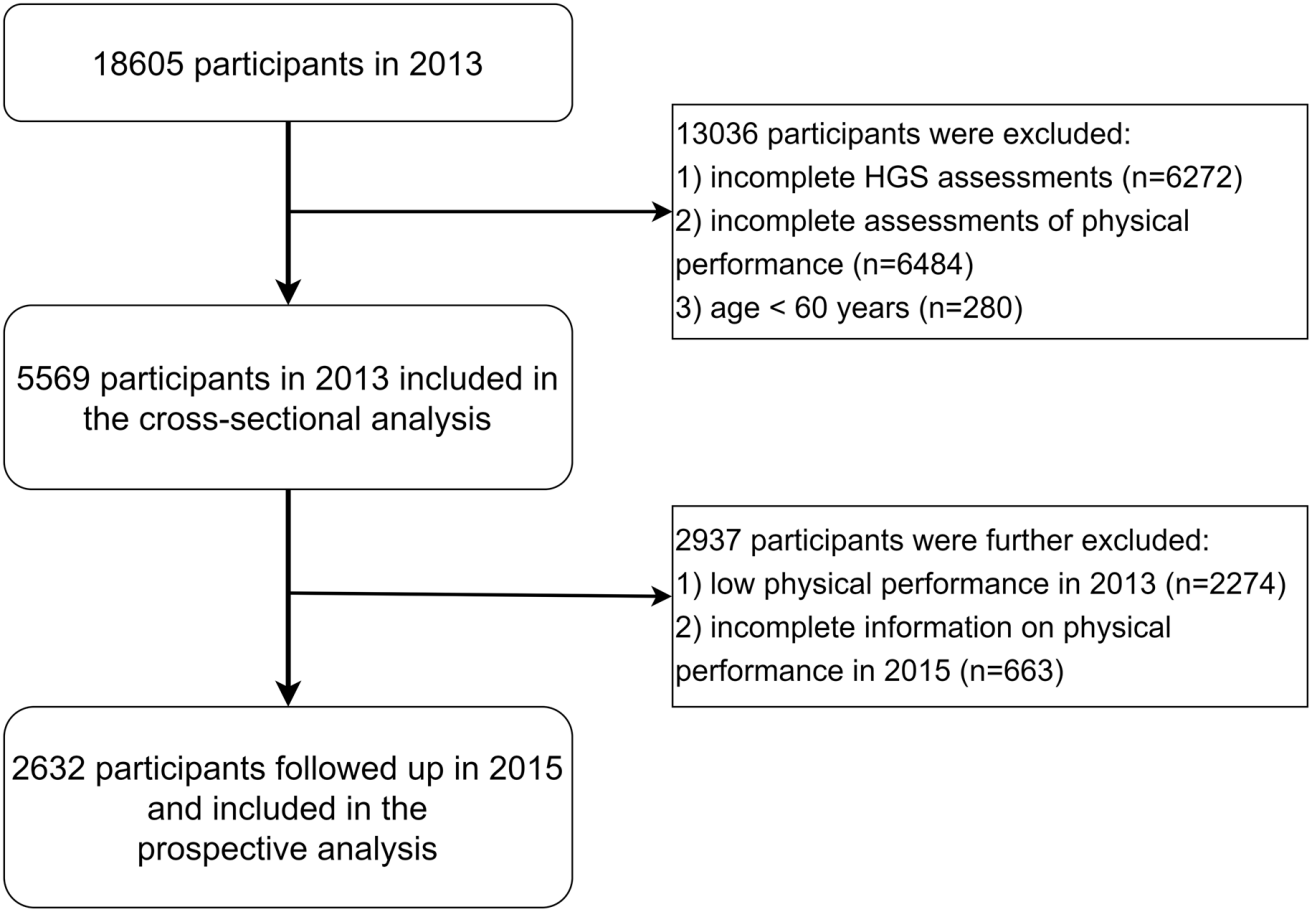
Flowchart of the sample selection process. *Abbreviations*: HGS, handgrip strength

This study used wave2 (2013) and wave3 (2015) data from the CHARLS dataset. In cross-sectional analysis, of the 18,605 participants in wave2, 13,036 were excluded based on the following exclusion criteria: (1) incomplete HGS measurements for both hands; (2) incomplete assessment of physical performance, including 5-times sit-to-stand test (5STS), gait speed, and the test of standing balance; (3) age < 60 years. In the longitudinal analysis, we further excluded the participants with a low physical performance in 2013 (wave 2) and missing data on physical performance in 2015 (wave3). The detailed flowchart of the sample selection process is depicted in Fig. 1. The definition of low physical performance will be provided in the “Physical Performance” section of the Methods. Finally, this research included 5,569 participants for the cross-sectional analysis and 2,632 for the longitudinal analysis.

### HGS weakness and asymmetry

HGS was measured using a mechanical dynamometer (Yuejian WL-1000, Nantong, China) by trained staff. According to the manufacturer, the precision of WL-1000 dynamometer was ± 3% (http://www.ntyuejian.com/3e/4.htm). Its test-retest reliability has been proven in previous research [18]. Participants were instructed to hold the dynamometer in a standing position with the elbow flexed at a 90° angle and squeeze the handle for a few seconds as hard as possible. This procedure was alternated between both hands and replicated twice for each hand. The greatest HGS value recorded across all four attempts was used to determine the maximal HGS of this participant. According to the Asian Work Group for Sarcopenia (AWGS) 2019 consensus, HGS weakness was defined as a maximal HGS of < 28 kg in older males and < 18 kg in older females [9]. The HGS ratio was computed by dividing the maximal HGS by the higher HGS value obtained from the opposite hand, ensuring that all HGS ratios were equal to or greater than 1.0 [22]. The HGS ratio can serve as an indicator of HGS asymmetry, with higher ones indicating more asymmetry (higher is worse). Previous studies usually used the “10% rule” to define HGS asymmetry, which meant that the larger HGS value of two hands was generally 10% greater than the smaller one [17, 24]. However, the precise criteria for HGS asymmetry remain to be conclusively established. Following published studies [14, 16], thresholds of 10%, 20%, and 30% disparity in muscle strength between hands were employed to categorize participants into HGS asymmetry or symmetry groups, with asymmetry defined as an HGS ratio exceeding 1.10, 1.20, or 1.30, according to the respective thresholds.

### HGS status

Both HGS weakness and asymmetry were abnormal conditions of muscle strength of the upper limbs. To elucidate the relationship between various HGS abnormalities (weakness, asymmetry, their coexistence) and physical performance, participants were stratified into four categories based on their HGS status in 2013 at baseline: (1) Normal (neither weakness nor asymmetry), (2) asymmetry only, (3) weakness only, and (4) Concurrent weakness and asymmetry [14]. Given the employment of three distinct thresholds for defining HGS asymmetry (HGS ratio > 1.10, > 1.20, and > 1.30), participants were correspondingly categorized based on these criteria to define their HGS status.

### Physical performance

The physical performance was evaluated by 5STS and SPPB. In 5STS, participants started in a seated position with arms folded across the chest and were instructed to stand up and sit back down again 5 times as quickly as possible. SPPB is a comprehensive assessment tools, consisting of three parts: 5STS, gait speed and the test of standing balance. For gait speed, each participant was asked to walk a 2.5-meter distance at a regular pace, doing this twice for a round trip. Time on the faster of two walks was used to calculate the gait speed. During the balance test, the participants were asked to maintain three stances for 10 s each: (1) side-by-side; (2) semi-tandem (the heel of one foot beside the big toe of the other); and (3) tandem (the heel of one foot in front of and touching the toes of the other). The sum of the scores from the three positions was used when calculating the SPPB score. The full score of SPPB was 12 points with 4 points for each test mentioned above and the detailed scoring criteria of SPPB are shown in Online Resource 1, referring to the previous research [1]. According to the AWGS 2019 consensus, low physical performance was defined as a 5STS time of ≥ 12 s, or an SPPB score of ≤ 9 [9]. The order of the three tests in SPPB was standing balance test first, then gait speed, and finally, the 5STS test, as described in previous literature [1].

This study refrained from using slow gait speed as an independent criterion for defining low physical performance, given the absence of a universally accepted threshold for slow gait speed over a 2.5-meter distance. The recommended cut-off values defining slow gait speed (0.8 m/s and 1.0 m/s) in the latest guideline for sarcopenia were all set based on longer distances of either 4–6 m [9, 10, 25]. Considering that most of the 2.5-meter walking distance is still in the initial acceleration phase, employing cut-off values of ≤ 0.8 m/s or ≤ 1.0 m/s to define slow gait speed may inadvertently underestimate the overall physical performance in this cohort, potentially leading to an overestimation of the prevalence of low physical performance among the older people. Additionally, the SPPB encompasses the measurement of gait speed. Therefore, older individuals who walked slowly markedly or failed to finish the walking—scoring 1 or 0 in the gait speed component of SPPB—would certainly meet the criteria for low physical performance, as indicated by an SPPB score of ≤ 9.

### Potential covariates

Drawing from previous knowledge, we also considered and adjusted for potential confounding factors in our study, including a range of sociodemographic characteristics and health-related factors. Sociodemographic characteristics included gender, age, residence (urban and rural), education level (illiteracy, elementary school and below, middle school, high school/vocational school, and bachelor’s degree/associate degree and above), marital status (married/cohabitated, widowed/divorced/separated and never married). As for health-related factors, we considered smoking status, drinking status, daily sleep time, cognitive function, and number of the 14 common chronic diseases (hypertension, dyslipidemia, diabetes or high blood sugar, cancer, chronic lung diseases, liver disease, heart disease, stroke, kidney disease, digestive system disease, emotional and psychiatric disease, memory-related disease, arthritis or rheumatism and asthma were included). In CHARLS wave2, the participants received assessment of cognitive function across four domains: orientation, memory, computation, and drawing. Orientation was measured by participants’ ability to identify the year, month, day, day of the week, and current season (1 point each, total 5 points). Computation was evaluated by participants’ capacity to consecutively subtract 7 from 100 five times (1 point per correct answer, total 5 points). Memory was assessed through delayed recall of ten randomly presented words (1 point per word recalled, total 10 points). Drawing ability was evaluated by participants’ replication of overlapping two pentagons (1 point for correct drawing). A total cognition score was calculated by summing scores across all domains (maximum 21 points). Body mass index (BMI) was calculated by dividing the weight (unit: kg) by the square of height (unit: m), and was further categorized as underweight (BMI < 18.5), normal (18.5 ≤ BMI < 24), overweight (24 ≤ BMI < 28), or obesity (BMI ≥ 28), according to China Working Group on Obesity [26]. Multiple imputations by chained equations with five imputations were used to impute missing covariate data [27], followed by a thorough comparative analysis to assess the consistency of the imputed data with the original dataset. The information of missing covariates and the results of the comparison are presented in Online Resource 2 and Online Resource 3, respectively. There were missing data for seven of the covariates we adjusted, and no significant difference exists between the data before and after multiple imputations.

### Statistical analysis

Quantitative data with a normal distribution were described as mean and standard deviation (SD) and, with non-normal distribution, as median and interquartile range (IQR). Categorical data were described using absolute and relative frequencies (%). Non-parametric Kruskal-Wallis test was used to evaluate the differences across the 4 groups (normal, asymmetry only, weakness only, and weakness and asymmetry). Post-hoc analysis was performed using the Dunn-Bonferroni pairwise comparison test. Percentages were compared by chi‐square test, with Bonferroni correction used for post hoc analysis.

The univariate and multivariate logistic regression model was employed to investigate the association between low physical performance and each of maximal HGS, HGS ratio, and HGS status, expressed in odds ratios (ORs) and 95% confidence intervals (CIs). Covariates adjusted in the multivariate logistic regression model included gender, age, residence, education level, marital status, smoking status, drinking status, daily sleep time, number of common chronic diseases, and BMI grade. The variance inflation factor (VIF) method was used to examine multicollinearity between the independent variables, and VIF ≥ 5 suggested multicollinearity in the model [28]. Results showed that no significant multicollinearity was found for all variables in our logistic regression analysis (data not shown). All statistical analyses were performed using RStudio Version 4.3.2, with the threshold for statistical significance set at a *p*-value of 0.05.

## Results

### Baseline characteristics of study population in 2013

The baseline characteristics of the participants by HGS status are listed in Table 1. Among the 5569 participants included in cross-sectional analysis, 2274 (40.83%) were classified into low physical performance group, while the remainder exhibited normal physical performance. The median age was 66.0 [63.0, 72.0] years and 51.4% were male. Defining HGS asymmetry as HGS ratio > 1.10, the prevalence of HGS asymmetry only, weakness only, and asymmetry and weakness together at baseline were 38.1%, 7.2%, and 7.6%, respectively.

Participants with HGS asymmetry only, weakness only, and two together had a higher proportion of impaired physical performance than those with neither (33.9% vs. 37.7%, 67.5%, and 73.6%, respectively). All the metrics of physical performance, including gait speed, 5STS time and balance test score, exhibited worsening trends from normal HGS to both asymmetry and weakness group (all, *p* < 0.001). The participants with weakness only, and asymmetry and weakness together were more likely to be older, rural residents, lower educated, widowed/divorced/separated, underweight, and exhibited lower cognitive scores.

**Table 1:** Baseline characteristics of study population by HGS status in 2013 CHARLS (*n* = 5569)

Characteristic	Overall (*n* = 5569)	Normal HGS (*n* = 2617)	Asymmetry only (*n* = 2124)	Weakness only (*n* = 403)	Weakness and asymmetry (*n* = 425)	*P*-Values
Gender, n (%)						
Male	2861 (51.4)	1385 (52.9)	1046 (49.2)	216 (53.6)	214 (50.4)	0.062
Female	2708 (48.6)	1232 (47.1)	1078 (50.8)	187 (46.4)	211 (49.6)	
Age, median (IQR)	66.0 (9.0)	66.0 (8.0) ^a^	66.0 (8.3) ^b^	73.0 (12.0) ^c^	73.0 (12.0) ^c^	< 0.001
Residence, n (%)						
Urban	1045 (18.8)	501 (19.1) ^a^	456 (21.5) ^a^	40 (9.9) ^b^	48 (11.3) ^b^	< 0.001
Rural	4524 (81.2)	2116 (80.9)	1668 (78.5)	363 (90.1)	377 (88.7)	
Education level, n (%)						
Illiteracy	1958 (35.2)	814 (31.1) ^a^	730 (34.4) ^a^	200 (49.6) ^b^	214 (50.4) ^b^	< 0.001
Elementary school and below	2577 (46.3)	1261 (48.2)	973 (45.8)	174 (43.2)	169 (39.8)	
Middle school	667 (12.0)	349 (13.3)	274 (12.9)	16 (4.0)	28 (6.6)	
High school/Vocational school	279 (5.0)	147 (5.6)	116 (5.5)	9 (2.2)	7 (1.6)	
Bachelor’s degree/Associate degree and above	88 (1.6)	46 (1.8)	31 (1.5)	4 (1.0)	7 (1.6)	
Marital status, n (%)						
Married/Cohabitated	4464 (80.2)	2201 (84.1) ^a^	1696 (79.8) ^b^	275 (68.2) ^c^	292 (68.7) ^c^	< 0.001
Widowed/Divorced/Separated	1063 (19.1)	400 (15.3)	412 (19.4)	123 (30.5)	128 (30.1)	
Never Married	42 (0.8)	16 (0.6)	16 (0.8)	5 (1.2)	5 (1.2)	
Smoking status, n (%)						
Never smoke	2972 (53.4)	1365 (52.2)	1177 (55.4)	204 (50.6)	226 (53.2)	0.096
Former/Current smoke	2597 (46.6)	1252 (47.8)	947 (44.6)	199 (49.4)	199 (46.8)	
Drinking status, n (%)						
Never drink	3002 (53.9)	1367 (52.2)	1163 (54.8)	233 (57.8)	239 (56.2)	0.073
Former/Current drink	2567 (46.1)	1250 (47.8)	961 (45.2)	170 (42.2)	186 (43.8)	
Daily sleep time, n (%)						
Normal (6–8 h/day)	2182 (39.2)	1060 (40.5) ^a^	839 (39.5) ^ab^	144 (35.7) ^ab^	139 (32.7) ^b^	0.001
Short (< 6 h/day)	1929 (34.6)	928 (35.5)	715 (33.7)	138 (34.2)	148 (34.8)	
Long (> 8 h/day)	1458 (26.2)	629 (24.0)	570 (26.8)	121 (30.0)	138 (32.5)	
BMI grade, n (%)						
Normal	2874 (51.6)	1309 (50.0) ^a^	1108 (52.2) ^a^	217 (53.8) ^b^	240 (56.5) ^b^	< 0.001
Underweight	484 (8.7)	191 (7.3)	160 (7.5)	67 (16.6)	66 (15.5)	
Overweight	1624 (29.2)	813 (31.1)	635 (29.9)	91 (22.6)	85 (20.0)	
Obesity	587 (10.5)	304 (11.6)	221 (10.4)	28 (6.9)	34 (8.0)	
Number of chronic diseases, median (IQR)	2.0 (2.0)	1.0 (2.0)	2.0 (2.0)	2.0 (2.0	2.0 (2.0)	0.202
Cognition score, median (IQR)	10.0 (8.0)	10.0 (7.0) ^a^	10.0 (7.0) ^a^	7.0 (8.0) ^b^	6.0 (9.0) ^b^	< 0.001
Gait speed, median (IQR)	0.73 (0.28)	0.76 (0.29) ^a^	0.73 (0.25) ^b^	0.61 (0.28) ^c^	0.59 (0.26) ^c^	< 0.001
5STS time, median (IQR)	10.34 (4.34)	9.94 (3.94) ^a^	10.15 (4.3) ^a^	12.52 (5.82) ^b^	13.09 (5.54) ^b^	< 0.001
Balance test score, n (%)						
0	37 (0.7)	12 (0.5) ^a^	2 (0.1) ^a^	10 (2.5) ^b^	13 (3.1) ^c^	< 0.001
1	72 (1.3)	20 (0.8)	24 (1.1)	12 (3.0)	16 (3.8)	
2	290 (5.2)	88 (3.4)	100 (4.7)	38 (9.4)	64 (15.1)	
3	261 (4.7)	106 (4.1)	99 (4.7)	27 (6.7)	29 (6.8)	
4	4909 (88.2)	2391 (91.4)	1899 (89.4)	316 (78.4)	303 (71.3)	
SPPB score, median (IQR)	11.0 (3.0)	11.0 (2.0) ^a^	11.0 (3.0) ^b^	9.0 (3.0) ^c^	9.0 (3.0) ^c^	< 0.001
Physical Performance in2013, n (%)						
Normal	3295 (59.2)	1729 (66.1) ^a^	1323 (62.3) ^b^	131 (32.5) ^c^	112 (26.4) ^c^	< 0.001
Low / Impaired	2274 (40.8)	888 (33.9)	801 (37.7)	272 (67.5)	313 (73.6)	

*Abbreviations* HGS, handgrip strength; BMI, body mass index; 5STS, 5-times sit-to-stand test; SPPB, short physical performance battery; SD, standard deviation; IQR, interquartile range. ^**a/b/c**^: Groups with the same letter are of the same subgroup in the post-hoc analysis

### Cross-sectional association between maximal HGS, HGS ratio and low physical performance in 2013

Both univariate and multivariate logistic regression analyses were adopted to investigate the cross-sectional associations between maximal HGS, HGS ratio, and low physical performance (Table 2). With each 1 kg increase in maximal HGS, the odds of low physical performance decreased by 7% in the crude model (OR 0.93, 95%CI 0.92–0.93). As for HGS asymmetry, every 0.10 (i.e., 10%) increase in HGS ratio was associated with 1.12-fold higher odds of low physical performance (95%CI: 1.08–1.16). These associations persisted in the fully-adjusted model 3, with an OR of 0.94 for each 1 kg augmentation in maximal HGS, and an OR of 1.10 for every 10% increase in HGS ratio.

**Table 2:** Cross-sectional association between Maximal HGS, HGS ratio and low physical performance in 2013 (*n* = 5569)

HGS parameters	Crude Model		Adjusted Model 1		Adjusted Model 2		Adjusted Model 3	
	OR (95% CI)	*P*-Values	OR (95% CI)	*P*-Values	OR (95% CI)	*P*-Values	OR (95% CI)	*P*-Values
Maximal HGS	0.93 (0.92–0.93)	< 0.001	0.94 (0.93–0.95)	< 0.001	0.94 (0.93–0.95)	< 0.001	0.94 (0.93–0.95)	< 0.001
HGS ratio (every 0.10 increase)	1.12 (1.08–1.16)	< 0.001	1.11 (1.07–1.15)	< 0.001	1.10 (1.06–1.15)	< 0.001	1.10 (1.06–1.15)	< 0.001

*Notes* Crude Model only includes Maximal HGS and HGS ratio;

Adjusted Model 1: crude model + gender, age, residence, education level, and marital status

Adjusted Model 2: model1 + smoking status, drinking status, and daily sleep time;

Adjusted Model 3: model2 + number of chronic diseases, cognition score and BMI grade

Abbreviations: HGS, handgrip strength; OR, odds ratio; CI, confidence interval

### Cross-sectional association between HGS Status and low physical performance in 2013

**Fig. 2:**
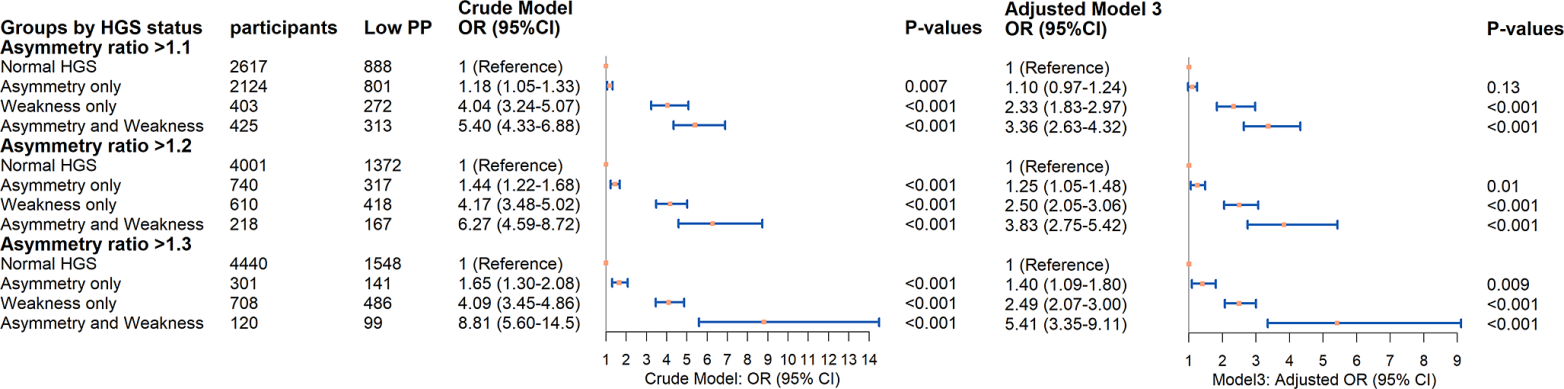
Forest Plots of Odds Ratios for Low Physical Performance in 2013, comparing between different HGS status in Crude Model and fully-adjusted Models 3 (*n* = 5569). *Notes*: Crude Model only includes HGS status; Adjusted Model 3: crude model + gender, age, residence, education level, marital status, smoking status, drinking status, daily sleep time, number of chronic diseases, cognition score and BMI grade; Abbreviations: HGS, handgrip strength; PP, physical performance; OR, odds ratio; CI, confidence interval

The cross-sectional associations between HGS status and low physical performance are shown in Fig. 2 and Online Resource 4. Compared with the normal groups, participants with HGS asymmetry only, weakness only, and both abnormalities showed a higher prevalence of low physical performance in the crude and adjusted models when the HGS asymmetry ratio was set at 20% and 30% thresholds (all, *p* < 0.001). After full adjustment in model 3, individuals with both abnormalities had the greatest odds of low physical performance (OR 3.83, 95% CI: 2.75–5.42 for the 20% threshold; OR 5.41, 95% CI: 3.35–9.11 for the 30% threshold). Participants with weakness only were associated with a 2.50-fold increase in the odds of low physical performance at 20% thresholds and a 2.49-fold increase at the 30% threshold. Those with asymmetry only had 1.25-fold and 1.40-fold higher odds at the 20% and 30% thresholds, respectively. At a 10% HGS asymmetry ratio, weakness only, and asymmetry and weakness together were significantly associated with a higher prevalence of low physical performance, even after full adjustment (weakness only: OR 2.33, 95% CI: 1.83–2.97; both: OR 3.36, 95% CI: 2.63–4.32). However, the association of asymmetry only with low physical performance was not observed in the adjusted models, despite being significant in the crude model.

### Prospective association between HGS status and low physical performance

**Fig. 3:**
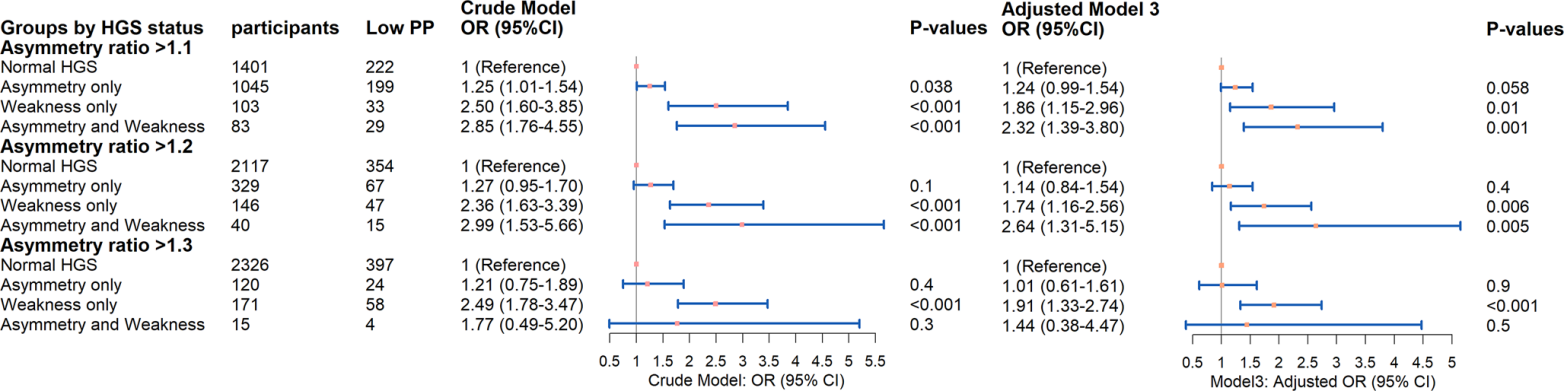
Forest Plots of Odds Ratios for new-onset Low Physical Performance in 2015, comparing between different baseline HGS status in Crude Model and fully-adjusted Models 3 (*n* = 2632). *Notes* Crude Model only includes HGS status; Adjusted Model 3: crude model + gender, age, residence, education level, marital status, smoking status, drinking status, daily sleep time, number of chronic diseases, cognition score and BMI grade; Abbreviations: HGS, handgrip strength; PP, physical performance; OR, odds ratio; CI, confidence interval

Among the 2632 individuals included in the longitudinal analysis, there were 483 (18.35%) older people who developed new-onset low physical performance after two years of follow-up. The prospective associations between HGS status in 2013 and low physical performance in 2015 are displayed in Fig. 3 and Online Resource 5. Weakness only was significantly associated with an increased risk of new-onset low physical performance, even after full adjustment (10% threshold: OR 1.86, 95% CI 1.15–2.96; 20% threshold: OR 1.74, 95% CI 1.16–2.56; 30% threshold: OR 1.91, 95% CI: 1.33–2.74). The addition of HGS asymmetry, defined by HGS ratios exceeding 1.1 and 1.2, to existing weakness, further increased the future risk of physical performance decline (10% threshold: OR 2.32, 95% CI 1.39–3.80; 20% threshold: OR 2.64, 95% CI 1.31–5.15).

## Discussion

To the best of our knowledge, this is the first study to investigate the associations of HGS weakness and asymmetry separately and together with physical performance within a nationally representative cohort of older Chinese adults. Our cross-sectional analysis highlighted that both HGS weakness and asymmetry correlated with low physical performance among the Chinese older people, in an additive manner. Additionally, longitudinal data suggested that older people with HGS weakness were at increased risk of developing new-onset low physical performance.

As underscored by a recent narrative review, previous research on the relationship between HGS value and physical performance in the older people shown mixed results, with relatively small sample size. While many studies found that the HGS value accurately reflected physical performance [29, 30], others reported little to no correlation, particularly concerning balance ability [21, 31, 32]. Our study, conducted within a large sample of the Chinese older population, confirmed the robust link between maximal HGS and physical performance. Specifically, the odds of low physical performance decreased by 7% in the crude model with each 1 kg increase in maximal HGS. Additionally, HGS weakness defined by AWGS 2019 consensus [9] was significantly associated with the current and future risk of low physical performance defined by 5STS and SPPB, thereby underscoring its effectiveness in detecting and predicting declines in physical performance among the older people.

The results derived from the cross-sectional analysis revealed that HGS asymmetry was associated with low physical performance, with every 10% increase in HGS ratio correlating with 12% greater odds for low physical performance. These results of our research are consistent with the previous research indicating that older Americans with HGS asymmetry were more likely to experience slow gait speed and poor standing balance [22]. From the perspective of neuroanatomy, HGS asymmetry may partially indicate the imbalanced activation of bilateral motor cortical areas and asymmetrical function of motor efferent pathways [33, 34], which could potentially impair balance function and mobility and impact the physical performance of the older people. Moreover, we also found that individuals with HGS weakness and asymmetry concurrently had greater odds of low physical performance than those with either HGS asymmetry or HGS weakness alone, with the odds escalating as the asymmetry ratio increasing. The results of previous research also suggested that presence of both HGS asymmetry and weakness together was more strongly associated with adverse health outcomes than each abnormality alone, such as motoric cognitive risk syndrome [14] and disability [17]. These findings highlighted the additive manner of how the two HGS abnormalities negatively influenced the health conditions correlated with muscle function among the older people.

One of the main strengths of this study is its pioneering role in exploring the association of HGS weakness and asymmetry simultaneously with physical performance in Chinese older people. Beyond cross-sectional analysis, we also conducted a longitudinal analysis to establish the predictive power of HGS abnormalities for physical performance decline over two years. Additionally, it offered novel evidence supporting the incorporation of HGS ratio calculation and asymmetry assessment into HGS protocols. Furthermore, the utilization of a nationally representative longitudinal survey of the Chinese older population significantly enhanced the reliability and generalizability of the findings.

However, several potential limitations of this study should be considered. Firstly, our definitions of low physical performance didn’t solely rely on slow gait speed. In the methodology section, it was explained that applying the thresholds of slow gait speed set for walking distances of four or six meters to a 2.5 m distance in CHARLS was deemed inappropriate. Nevertheless, since gait speed was already a component of the SPPB, individuals who performed poorly in this part would still be classified as having low physical performance based on the SPPB standard. This conservative approach was taken to prevent overestimation of the proportion of older people with low physical performance. Secondly, while this research has accounted for various potential confounding factors, it should be noted that other potential influencers, like physical activity and dietary patterns, were not included. Lastly, the data obtained from questionnaires were self-reported, which introduces the possibility of recall bias and self-representation bias that should be acknowledged.

In conclusion, this research highlighted the cross-sectional and prospective associations of HGS weakness and asymmetry with low physical performance in the Chinese older people, underscoring the significance of HGS measurement as a tool for identifying and predicting decline in physical performance. Given that HGS is a good marker of physical performance, the HGS test, which is simpler, more cost-effective and feasible to conduct, can be considered as an alternative method for assessing physical performance, especially in some clinical settings. When evaluating older people, such as during comprehensive geriatric assessments (CGA), healthcare professionals should consider assessing HGS asymmetry in addition to HGS weakness. Our study will help healthcare professionals, especially those in primary care, screen older individuals with low physical performance more efficiently, and identify those at higher risk of developing new-onset physical performance decline within two years. This will further allow for early risk assessment and education interventions concerning adverse events such as falls, fractures, and disability, followed by implementation of preventive interventions including exercise training. The findings of this research must be confirmed in different populations, and additional research is needed to explore the associations between HGS and other measures of physical performance.

## Electronic supplementary material

Below is the link to the electronic supplementary material.

Supplementary Material 1

Supplementary Material 2

Supplementary Material 3

Supplementary Material 4

Supplementary Material 5

## Data Availability

Access to CHARLS datasets is facilitated through its official website at: https://charls.charlsdata.com/pages/Data/2013-charls-wave2/zh-cn.html.
