# Supplementary material for: Association of handgrip strength weakness and asymmetry with low physical performance among Chinese older people
Source: Aging Clin Exp Res. 2024 Nov 25;36(1):225. doi: 10.1007/s40520-024-02886-5 (PMC11588951; doi:10.1007/s40520-024-02886-5)
Supplement: Supplementary file 1 — Supplementary Material 1 [file 40520_2024_2886_MOESM1_ESM.docx]

**ESM_1 Scoring criteria of each component in SPPB**^1^

| Test | Performance in the test | Score |
| --- | --- | --- |
| Balance test* | | |
| (1): Side-by-side stand | held for 10 seconds | 1 point |
|  | not held for 10 seconds | 0 points |
|  | not attempted | 0 points |
| (2): Semi-tandem stand | held for 10 seconds | 1 point |
|  | not held for 10 seconds | 0 points |
|  | not attempted | 0 points |
| (3): Tandem stand | held for 10 seconds | 2 points |
|  | held for 3-9.99 seconds | 1 point |
|  | not held for <3 seconds | 0 points |
|  | not attempted | 0 points |
| Gait speed | unable to complete | 0 points |
|  | ≤0.43 m/s | 1 point |
|  | 0.43-0.60 m/s | 2 points |
|  | 0.60-0.77 m/s | 3 points |
|  | ＞0.77m/s | 4 points |
| 5-times sit-to-stand test (5STS) | >60 seconds or unable to complete | 0 points |
|  | 16.6-60 seconds | 1 point |
|  | 13.6-16.6 seconds | 2 points |
|  | 11.1-13.6 seconds | 3 points |
|  | ≤ 11.1 seconds | 4 points |
| *Noted*: *In the balance test, the sum of the scores from the three positions was used to determine the test score, which was further utilized to calculate the SPPB score.  Abbreviations: SPPB, short physical performance battery. | | |

**References:**

**1.** Guralnik JM, Ferrucci L, Pieper CF, Leveille SG, Markides KS, Ostir GV, et al. Lower extremity function and subsequent disability: consistency across studies, predictive models, and value of gait speed alone compared with the short physical performance battery. *J Gerontol A Biol Sci Med Sci.* 2000;55(4):M221-M231.
