# Supplementary material for: Association of handgrip strength weakness and asymmetry with low physical performance among Chinese older people
Source: Aging Clin Exp Res. 2024 Nov 25;36(1):225. doi: 10.1007/s40520-024-02886-5 (PMC11588951; doi:10.1007/s40520-024-02886-5)
Supplement: Supplementary file 2 — Supplementary Material 2 [file 40520_2024_2886_MOESM2_ESM.docx]

**ESM_2 Information of missing covariates at baseline** **in 2013 CHARLS**

| Covariates | Missing | Total | Missing percentage |
| --- | --- | --- | --- |
| Residence | 292 | 5569 | 5.24% |
| Education level | 280 | 5569 | 5.03% |
| Smoking status | 7 | 5569 | 0.13% |
| Drinking status | 10 | 5569 | 0.18% |
| Daily sleep time | 347 | 5569 | 6.23% |
| BMI grade | 42 | 5569 | 0.75% |
| Number of chronic diseases | 133 | 5569 | 2.39% |
| Cognition score | 574 | 5569 | 10.31% |
