# Supplementary material for: Association of handgrip strength weakness and asymmetry with low physical performance among Chinese older people
Source: Aging Clin Exp Res. 2024 Nov 25;36(1):225. doi: 10.1007/s40520-024-02886-5 (PMC11588951; doi:10.1007/s40520-024-02886-5)
Supplement: Supplementary file 3 — Supplementary Material 3 [file 40520_2024_2886_MOESM3_ESM.docx]

**ESM_3 Comparison of general characteristics before and after Multiple Imputations**

| Characteristic | Before | After | *P-*Values |
| --- | --- | --- | --- |
| Residence, n (%) |  |  |  |
| Urban | 959 (18.2) | 1045 (18.8) | 0.442 |
| Rural | 4318 (81.8) | 4524 (81.2) |  |
| Education level, n (%) |  |  |  |
| Illiteracy | 1836 (34.7) | 1958 (35.2) | 0.988 |
| Elementary school and below | 2459 (46.5) | 2577 (46.3) |  |
| Middle school | 646 (12.2) | 667 (12.0) |  |
| High school/Vocational school | 266 (5.0) | 279 (5.0) |  |
| Bachelor’s degree/Associate degree and above | 82 (1.6) | 88 (1.6) |  |
| Smoking status, n (%) |  |  |  |
| Never smoke | 2967 (53.3) | 2972 (53.4) | 0.996 |
| Former/Current smoke | 2595 (46.7) | 2597 (46.6) |  |
| Drinking status, n (%) |  |  |  |
| Never drink | 2996 (53.9) | 3002 (53.9) | 1 |
| Former/Current drink | 2563 (46.1) | 2567 (46.1) |  |
| Daily sleep time, n (%) |  |  |  |
| Normal (6-8h/day) | 2013 (38.5) | 2182 (39.2) | 0.302 |
| Short (<6h/day) | 1882 (36.0) | 1929 (34.6) |  |
| Long (>8h/day) | 1327 (25.4) | 1458 (26.2) |  |
| BMI grade, n (%) |  |  |  |
| Normal | 2866 (51.9) | 2874 (51.6) | 0.846 |
| Underweight | 454 (8.2) | 484 (8.7) |  |
| Overweight | 1622 (29.3) | 1624 (29.2) |  |
| Obesity | 585 (10.6) | 587 (10.5) |  |
| Number of chronic diseases, median (IQR) | 1.0 (2.0) | 2.0 (2.0) | 0.365 |
| Cognition score (IQR) | 10.0 (7.0) | 10.0 (8.0) | 0.201 |

Abbreviations: IQR, interquartile range.
