# Supplementary material for: Association of handgrip strength weakness and asymmetry with low physical performance among Chinese older people
Source: Aging Clin Exp Res. 2024 Nov 25;36(1):225. doi: 10.1007/s40520-024-02886-5 (PMC11588951; doi:10.1007/s40520-024-02886-5)
Supplement: Supplementary file 4 — Supplementary Material 4 [file 40520_2024_2886_MOESM4_ESM.docx]

**ESM_4 Cross-sectional association between HGS Status and Low Physical Performance in 2013 CHARLS (n=5569)**

| HGS Status defined by different asymmetry ratio | Crude Model | | Model 1 | | Model 2 | | Model 3 | |
| --- | --- | --- | --- | --- | --- | --- | --- | --- |
|  | **OR (95% CI)** | ***p* Value** | **OR (95% CI)** | ***p* Value** | **OR (95% CI)** | ***p* Value** | **OR (95% CI)** | ***p* Value** |
| HGS asymmetry ratio >1.1 | | | | | | | | |
| Neither weakness nor asymmetry (n=2617) | reference | NA | reference | NA | reference | NA | reference | NA |
| Asymmetry only (n=2124) | 1.18 (1.05-1.33) | 0.007 | 1.10 (0.98-1.25) | 0.12 | 1.10 (0.97-1.25) | 0.13 | 1.10 (0.97-1.24) | 0.13 |
| Weakness only (n=403) | 4.04 (3.24-5.07) | <0.001 | 2.63 (2.08-3.35) | <0.001 | 2.62 (2.06-3.33) | <0.001 | 2.33 (1.83-2.97) | <0.001 |
| Weakness and asymmetry (n=425) | 5.4 (4.33-6.88) | <0.001 | 3.67 (2.88-4.69) | <0.001 | 3.63 (2.85-4.64) | <0.001 | 3.36 (2.63-4.32) | <0.001 |
| HGS asymmetry ratio >1.2 | | | | | | | | |
| Neither weakness nor asymmetry (n=4001) | reference | NA | reference | NA | reference | NA | reference | NA |
| Asymmetry only (n=740) | 1.44 (1.22-1.68) | <0.001 | 1.29 (1.09-1.53) | 0.003 | 1.29 (1.09-1.53) | 0.003 | 1.25 (1.05-1.48) | 0.010 |
| Weakness only (n=610) | 4.17 (3.48-5.02) | <0.001 | 2.81 (2.31-3.42) | <0.001 | 2.79 (2.30-3.40) | <0.001 | 2.50 (2.05-3.06) | <0.001 |
| Weakness and asymmetry (n=218) | 6.27 (4.59-8.72) | <0.001 | 4.21 (3.03-5.93) | <0.001 | 4.18 (3.01-5.89) | <0.001 | 3.83 (2.75-5.42) | <0.001 |
| HGS asymmetry ratio >1.3 | | | | | | | | |
| Neither weakness nor asymmetry (n=4440) | reference | NA | reference | NA | reference | NA | reference | NA |
| Asymmetry only (n=301) | 1.65 (1.30-2.08) | <0.001 | 1.45 (1.14-1.86) | 0.003 | 1.44 (1.13-1.84) | 0.004 | 1.40 (1.09-1.80) | 0.009 |
| Weakness only (n=708) | 4.09 (3.45-4.86) | <0.001 | 2.80 (2.33-3.36) | <0.001 | 2.78 (2.32-3.34) | <0.001 | 2.49 (2.07-3.00) | <0.001 |
| Weakness and asymmetry (n=120) | 8.81 (5.60-14.5) | <0.001 | 5.68 (3.54-9.51) | <0.001 | 5.62 (3.50-9.42) | <0.001 | 5.41 (3.35-9.11) | <0.001 |

*Notes*: Crude Model only includes HGS status;

Model 1: crude model + gender, age, residence, education level, and marital status;

Model 2: model1 + smoking status, drinking status, and daily sleep time;

Model 3: model2 + number of chronic diseases, cognition score and BMI grade;

Abbreviations: HGS, handgrip strength; OR, odds ratio; CI, confidence interval.
