# Supplementary material for: Association of handgrip strength weakness and asymmetry with low physical performance among Chinese older people
Source: Aging Clin Exp Res. 2024 Nov 25;36(1):225. doi: 10.1007/s40520-024-02886-5 (PMC11588951; doi:10.1007/s40520-024-02886-5)
Supplement: Supplementary file 5 — Supplementary Material 5 [file 40520_2024_2886_MOESM5_ESM.docx]

**ESM_5 Prospective association between baseline HGS Status in 2013 and Low Physical Performance in 2015 (n=2632)**

| HGS Status defined by different asymmetry ratio | Crude Model | | Model 1 | | Model 2 | | Model 3 | |
| --- | --- | --- | --- | --- | --- | --- | --- | --- |
|  | **OR (95% CI)** | ***p* Value** | **OR (95% CI)** | ***p* Value** | **OR (95% CI)** | ***p* Value** | **OR (95% CI)** | ***p* Value** |
| HGS asymmetry ratio >1.1 | | | | | | | | |
| Neither weakness nor asymmetry (n=1401) | reference | NA | reference | NA | reference | NA | reference | NA |
| Asymmetry only (n=1045) | 1.25 (1.01-1.54) | 0.038 | 1.20 (0.97-1.49) | 0.092 | 1.22 (0.98-1.51) | 0.076 | 1.24 (0.99-1.54) | 0.058 |
| Weakness only (n=103) | 2.50 (1.60-3.85) | <0.001 | 1.92 (1.20-3.02) | 0.006 | 1.96 (1.22-3.10) | 0.004 | 1.86 (1.15-2.96) | 0.010 |
| Weakness and asymmetry (n=83) | 2.85 (1.76-4.55) | <0.001 | 2.20 (1.33-3.58) | 0.002 | 2.25 (1.36-3.66) | 0.001 | 2.32 (1.39-3.80) | 0.001 |
| HGS asymmetry ratio >1.2 | | | | | | | | |
| Neither weakness nor asymmetry (n=2117) | reference | NA | reference | NA | reference | NA | reference | NA |
| Asymmetry only (n=329) | 1.27 (0.95-1.70) | 0.1 | 1.15 (0.85-1.54) | 0.4 | 1.17 (0.86-1.57) | 0.3 | 1.14 (0.84-1.54) | 0.4 |
| Weakness only (n=146) | 2.36 (1.63-3.39) | <0.001 | 1.79 (1.20-2.62) | 0.003 | 1.81 (1.22-2.66) | 0.003 | 1.74 (1.16-2.56) | 0.006 |
| Weakness and asymmetry (n=40) | 2.99 (1.53-5.66) | <0.001 | 2.44 (1.22-4.72) | 0.009 | 2.53 (1.26-4.91) | 0.007 | 2.64 (1.31-5.15) | 0.005 |
| HGS asymmetry ratio >1.3 | | | | | | | | |
| Neither weakness nor asymmetry (n=2326) | reference | NA | reference | NA | reference | NA | reference | NA |
| Asymmetry only (n=120) | 1.21 (0.75-1.89) | 0.4 | 1.08 (0.66-1.70) | 0.8 | 1.07 (0.65-1.69) | 0.8 | 1.01 (0.61-1.61) | 0.9 |
| Weakness only (n=171) | 2.49 (1.78-3.47) | <0.001 | 1.96 (1.36-2.78) | <0.001 | 1.99 (1.39-2.83) | <0.001 | 1.91 (1.33-2.74) | <0.001 |
| Weakness and asymmetry (n=15) | 1.77 (0.49-5.20) | 0.3 | 1.24 (0.33-3.79) | 0.7 | 1.26 (0.33-3.87) | 0.7 | 1.44 (0.38-4.47) | 0.5 |

Notes: Crude Model only includes HGS status;

Model 1: crude model + gender, age, residence, education level, and marital status;

Model 2: model1 + smoking status, drinking status, and daily sleep time;

Model 3: model2 + number of chronic diseases, cognition score and BMI grade;

Abbreviations: HGS, handgrip strength; OR, odds ratio; CI, confidence interval.
